# The utility of biopsy in pyoderma gangrenosum: a retrospective cohort study

**DOI:** 10.1093/skinhd/vzaf087

**Published:** 2026-01-09

**Authors:** Angel M Moore, Jamie L Karch, Katherine E Bradley, Mirjana Stevanovic, Iman Salem, Dylan J Parker, Brian J Simmons

**Affiliations:** Dartmouth College, Geisel School of Medicine, Hanover, NH, USA; Department of Dermatology, Dartmouth College, Dartmouth Hitchcock Medical Center, Lebanon, NH, USA; Dartmouth College, Geisel School of Medicine, Hanover, NH, USA; Dartmouth College, Geisel School of Medicine, Hanover, NH, USA; Department of Dermatology, Dartmouth College, Dartmouth Hitchcock Medical Center, Lebanon, NH, USA; Department of Dermatology, Dartmouth College, Dartmouth Hitchcock Medical Center, Lebanon, NH, USA; Dartmouth College, Geisel School of Medicine, Hanover, NH, USA; Department of Dermatology, Dartmouth College, Dartmouth Hitchcock Medical Center, Lebanon, NH, USA

## Abstract

**Background:**

Pyoderma gangrenosum (PG) is a rare skin condition characterized by chronic, painful ulcerations. It is considered a diagnosis of exclusion, often resulting in diagnostic delay. Histological results in PG, even when biopsied despite a risk of pathergy, are nonspecific.

**Objectives:**

To examine retrospectively the utility of biopsies in the diagnosis of 58 patients treated for PG.

**Methods:**

Medical records were reviewed to compare biopsy results versus the efficacy of diagnostic rating systems (Delphi, Su, PARACELSUS).

**Results:**

Among 58 patients, 26 (45%) underwent biopsies, with only 10 (38%) contributing to a PG diagnosis. As a single-centre and retrospective study, documentation style and sample size limit generalizability.

**Conclusions:**

Given risk of pathergy, nonspecific histopathological findings and low sensitivity, in our opinion, based on this small sample size, biopsies have limited diagnostic value for PG.

## Teaser

Three scoring tools used for the diagnosis of pyoderma gangrenosum are compared and the role of biopsy in pyoderma gangrenosum diagnosis is examined using 51 cases. Biopsies have limited diagnostic value in pyoderma gangrenosum, while the PARACELSUS scoring tool has the greatest sensitivity for diagnosing pyoderma gangrenosum.

What is already known about this topic?

- The diagnosis of pyoderma gangrenosum is difficult.
- Three scoring tools are available for the diagnosis of pyoderma gangrenosum: PARACELSUS, Su and Delphi.
- The utility of each method and the role of biopsy in the accurate diagnosis of pyoderma gangrenosum remains unclear.

What does this study add?

- Comparing each of these scoring systems using clinical cases in which biopsies were and were not provided, the present study provides additional clarity that biopsies have limited diagnostic value in pyoderma gangrenosum.
- The PARACELSUS scoring tool has the greatest sensitivity for diagnosing pyoderma gangrenosum.

Pyoderma gangrenosum (PG) poses significant challenges when making a definitive diagnosis and is commonly considered a diagnosis of exclusion. Currently, there are three diagnostic rating tools available for this purpose: the Delphi method (sensitivity and specificity of 86% and 90%, respectively), the Su method (specific sensitivity and specificity data remain unreported in the existing literature) and PARACELSUS (sensitivity and specificity values of 100% and 96%, respectively).^1,2^

One of the key criteria within the Delphi method involves the requirement for a biopsy with neutrophilic infiltrate. However, the diagnostic utility of biopsy in the context of PG remains a subject of debate, given the associated risk of pathergy and the absence of histopathological findings that are pathognomonic for PG.^3^ The main objective of this study was to evaluate the utility of these diagnostic surveys in a retrospective analysis of three PG diagnostic rating tools on patients with suspected PG. We also examined the rate and histopathological findings of biopsies obtained to determine their usefulness in the diagnosis of PG.

## Materials and methods

A retrospective review of electronic medical records was conducted to identify all adult patients with an initial diagnostic suspicion of PG based upon clinical exam who presented to a Northeastern academic referral centre between 2013 and 2023. Fifty-eight patients were identified with diagnoses of PG. Six of these patients went on to receive alternative diagnoses and one patient had insufficient data to apply the PG diagnostic rating criteria, and were therefore considered to be false positives and omitted from individual diagnostic criteria calculations. Three investigators manually reviewed electronic medical records to determine scores for Delphi, Su and PARACELSUS for these 58 patients, to assess tool sensitivity and specificity. Biopsy results, histopathological descriptions and providers’ comments on the utility of biopsy results from chart review were also extracted from the records of the 58 patients treated for PG. A patient case biopsy was deemed contributory in diagnosis only if there was written documentation in the electronic medical record indicating that the provider referenced biopsy in their medical decision-making rationale in favour of a diagnosis of PG, within either an encounter note or recorded updates or communications. A biopsy was considered noncontributory if there was no documentation explicitly referencing the role of biopsy in aiding diagnosis.

## Results

Of the 58 patients diagnosed with PG included in this study, 26 (45%) underwent biopsy. A total of 32 patients (55%) did not undergo biopsy due to shared decision-making or concern of pathergy, and were diagnosed based on clinical observation alone. Among the 26 biopsies conducted, 10 (38%) were considered contributory in confirming the PG diagnosis, while the remaining 16 (62%) were considered noncontributory, as defined by the parameters above. Seventy per cent of the 10 patients whose biopsies were contributory in their diagnosis of PG had neutrophilic infiltration. In applying diagnostic frameworks to patients who underwent biopsy, PARACELSUS yielded a sensitivity of 96%, while specificity was 33%. Su and Delphi criteria yielded 100% specificity, but 65% and 19% sensitivities, respectively. In the nonbiopsy group, PARACELSUS revealed a sensitivity and specificity of 100%. The Su criteria also demonstrated a specificity of 100%, but sensitivity was 12% (Table 1).

**Table 1:** Test performance characteristics of the PARACELSUS score, Su criteria and Delphi criteria

	Biopsy (*n* = 26)			No biopsy^a^ (*n* = 32)	
	Delphi	PARACELSUS	Su	PARACELSUS	Su
Sensitivity	0.187	0.961	0.653	1	0.120
Specificity	1	0.333	1	1	1
Positive predictive value	1	0.925	1	1	1
Negative predictive value	0.130	0.5	0.150	1	0.153

^a^Delphi criteria not applied to patients where no biopsy was obtained as a major criterion requires the presence of a biopsy.

## Discussion

The pathogenesis of PG is yet to be fully understood, and is currently considered a rare, neutrophilic, inflammatory dermatosis.^1^ Our results highlight the limited utility of relying on the findings of neutrophilic infiltration on biopsy, instead drawing attention to reliance on clinical criteria and scoring methods for diagnosis of PG. It was previously reported that when all three PG diagnostic tools were retrospectively applied to a cohort of patients with PG, the PARACELSUS score correctly identified the highest proportion of patients with PG (89%), and the Su and Delphi criteria both identified 74% of patients.^2^ The PARACELSUS tool is reported to yield a sensitivity of 100% with a score ≥10, which closely reflects the results in our cohort, demonstrating sensitivities of 96% and 100% in the biopsy and nonbiopsy groups, respectively.^2^ Of all three diagnostic tools, we found PARACELSUS to be the tool with greatest sensitivity for diagnosing PG in our cohort, both with and without biopsy. This scoring method hinges on common PG clinical characteristics and observation of the ulceration as major criteria: progressive course of disease, absence of relevant differential diagnosis and reddish-violaceous border (Table 2). Notably, and unlike other PG diagnostic tools, a biopsy is only a minor criterion for the PARACELSUS score, highlighting that the clinical scenario is overall a better diagnostic predictor than histopathology especially in the context of inconsistent rates of diagnostic biopsy in PG. This idea is also reflected in our reported PARACELSUS specificity, which ranged from 33% in the biopsy group to 100% in the nonbiopsy group, suggesting reduced ability in identifying true negatives when a biopsy is involved, perhaps because biopsy results, given their nonspecific nature, may limit clinicians’ willingness to designate a sole underlying diagnosis. The Su criteria demonstrated the second highest sensitivities at 65% in the biopsy group and 12% in the nonbiopsy group. Specificity was 100%. This diagnostic framework bases its major criteria on similar clinical characteristic and lesion progression as the PARACELSUS score, but the Su framework also poses that the ulcer must be painful, necrolytic and have an undermined border to meet the criteria (Table 3). This perhaps limits otherwise positive results, as not all PG ulcerations in our cohort concurrently had all three variables of a painful, necrolytic and undermined border. Additionally, a minor criterion for Su requires histopathological findings, which reduces the sensitivity of the rating tool on patient cases where biopsies are not obtained, as shown with the increased sensitivity among the biopsy group. Yet, even with a biopsy, the sensitivity falls short of PARACELSUS sensitivities with or without a biopsy.

**Table 2:** The PARACELSUS score: individual diagnostic criteria and occurrence in patients with pyoderma gangrenosum (*n* = 51)

	Occurrence, n (%)
Progressive course of disease (<6 weeks) (3)	34 (66)
Absence of relevant differential diagnosis (3)	41 (80)
Reddish-violaceous wound border (3)	51 (100)
Amelioration due to systematic treatment to first-line treatment (steroids or biologics) (2)	41 (80)
Extreme pain >4 (2)	43 (84)
Localized pathergy phenomenon (2)	39 (76)
Characteristically bizarre ulcer shape (2)	51 (100)
Suppurative inflammation on histopathology (1)	5 (10)
Undermined wound margin (1)	39 (76)
Associated systematic disease (1) (IBD, IgA gammopathy, underlying malignancy, rheumatoid arthritis)	24 (47)

Points for each criterion are given in parentheses. The number of patients totalled 51 as 7 patients were excluded as explained in the main text. IBD, inflammatory bowel disease.

**Table 3:** The Su criteria: individual diagnostic criteria and occurrence in patients with pyoderma gangrenosum (*n* = 51)

	Occurrence (%)
Rapid progression of painful, necrolytic cutaneous ulcer with an irregular, violaceous and undermined border	21 (41)
Other causes of cutaneous ulceration have been excluded	41 (80)
History suggestive of pathergy or clinical finding of cribriform scarring	43 (84)
Systemic disease associated with PG (IBD, IgA gammopathy, underlying malignancy, rheumatoid arthritis)	24 (47)
Histopathological findings (sterile dermal neutrophilia ± mixed inflammation ± lymphocytic vasculitis)	17 (33)
Treatment response (50% reduction of size within 1 month to systemic steroid treatment)	11 (22)

The number of patients totalled 51 as 7 patients were excluded as explained in the main text. IBD, inflammatory bowel disease.

The Delphi method is reported to yield a sensitivity of 86% and specificity of 90%. We report a sensitivity of 18% and specificity of 100% when applied to the biopsy group. The major ­criterion for this scoring method, unlike the previous two methods discussed, is a biopsy from the ulcer edge with neutrophilic infiltrate, and, therefore, this framework was not applied to the nonbiopsy group (Table 4). Only about half of patients who had a positive diagnosis for PG in our cohort underwent biopsy, and only 7 of the 26 (27%) biopsies obtained met the major criterion of neutrophilic infiltrate. The sensitivity of the Delphi system is lower than that of the PARACELSUS and Su systems, regardless of whether a biopsy was performed. One reason why Delphi may have limited use is because the panel recommended biopsy as a requirement in the diagnosis of PG with a major criterion of neutrophilic inflammation. However, there are no reported definitive histological findings specific for PG.^3^ The ‘characteristic’ histological findings of PG are dermal neutrophilic infiltration, followed by nonspecific epidermal necrosis and ulceration.^4^ However, neutrophilic infiltrate is found mainly in acute stages of PG, and chronic ulcerations might have additional histopathological findings (e.g. lymphohistiocytic infiltrate). For example, a retrospective review from 2011 demonstrated that only 7% (*n* = 8/103) of patients with PG diagnosis had characteristic biopsy findings of neutrophilic infiltrate and early abscess formation.^2^ Additionally, neutrophilic infiltration is also a classic finding of a chronic ulcer, highlighting the nonspecific nature of histological findings.^5^ The majority of our patients who were diagnosed with PG and underwent biopsy did not have classic findings of neutrophilic infiltration (42%). Our study demonstrates that setting nonspecific histopathological findings as a major criterion for PG limits the sensitivity of diagnostic rating tools for PG.^6^ The Delphi criteria underscore the controversy surrounding the utility of biopsy in diagnosing PG. Biopsy can be necessary to rule out other concerning potential diagnoses on the differential including infection, vasculitis or malignancy, especially if the patient presentation and lesion slightly vary from classic PG. Specifically, one patient in this study had a progressively worsening acute-on-chronic 20-year ulcer that was thought to be PG based on the clinical context and was later diagnosed as basal cell carcinoma by biopsy. Therefore, biopsy still retains utility in cases where a range of differential diagnoses are plausible. However, providers should be judicious with biopsy due to the risk of pathergy in PG, wherein the trauma to the skin induces an overreactive inflammatory process that has the potential to exacerbate the disease.^5^ Studies have shown that the risk of pathergy can occur in approximately 25% of patients with minor trauma.^4^ However, if pathergy does occur with biopsy, it can be applied to the diagnosis of PG as it is a minor criterion for all three scoring methods.

**Table 4:** The Delphi criteria: individual diagnostic criteria and occurrence in patients with pyoderma gangrenosum (*n* = 51)

	Occurrence (%)
Biopsy with neutrophilic infiltrate	9 (18)
Exclusion of infection on histology	21 (41)
Pathergy	37 (73)
History of IBD or inflammatory arthritis	14 (27)
Papule, pustule or vesicle that rapidly ulcerates	18 (35)
Peripheral erythema, undermining border and tenderness at the site of ulceration	34 (67)
Multiple ulcerations (at least one occurring on an anterior lower leg)	27 (53)
Cribriform or wrinkled paper scars at healed ulcer sites	33 (65)
Decrease in ulcer size after immunosuppressive treatment within 1 month	24 (47)

The number of patients totalled 51 as 7 patients were excluded as explained in the main text. IBD, inflammatory bowel disease.

In conclusion, our study highlights PARACELSUS as the preferred diagnostic tool when compared with the Delphi and Su criteria. Our findings emphasize the importance of clinical history and close follow-up in diagnosing PG. Our data suggest that while biopsy holds limited diagnostic value in confirming PG, its role remains crucial for atypical presentations of PG where plausible differential diagnoses should be excluded. In rare cases, biopsies are able to provide data to make a critical alternative diagnosis, such as the presence of malignant lesions. Moving forward, further research may focus on redefining the PG diagnosis from an exclusion-based approach to one rooted in a gold-standard diagnostic framework, potentially by integrating the PARACELSUS method. Limitations of this study include its retrospective design, in which clinical notes, clinical photographs and pathology reports were used for analysis. As a result, physicians were not directly asked for their opinions regarding the utility of biopsy in PG. Consequently, a negative or noncontributory biopsy recorded in our review may well have been discussed during the case but not documented in the medical record. This highlights the need for future studies to actively engage physicians and capture their perspectives on the utility of biopsy in PG. Furthermore, this study was performed at a single clinical site.

## Supplementary Material

vzaf087_Supplementary_Data

## Data Availability

Delphi criteria, PARACELSUS scoring, Su criteria, biopsy collection and the histological findings of the cohort of 58 patients studied are provided in Table S1 (see Supporting Information).
