# Supplementary material for: The utility of biopsy in pyoderma gangrenosum: a retrospective cohort study
Source: Skin Health Dis. 2026 Jan 9;6(2):90–3. doi: 10.1093/skinhd/vzaf087 (PMC13036715; doi:10.1093/skinhd/vzaf087)
Supplement: vzaf087_Supplementary_Data [file vzaf087_supplementary_data.docx]

Supplemental Table 1. Delphi criteria, PARACELSUS scoring, Su criteria, biopsy collection, and histologic findings of a cohort of 58 patients

| Patient | Delphi Criteria Met | PARACELSUS Score | Su Criteria Met | Biopsy Completed Yes (Y) or No (N) | Biopsy Contributory Yes (Y) or No (N) | Histologic Features of Biopsy | Diagnosis Changed from PG Yes (Y) or N (N) |
| --- | --- | --- | --- | --- | --- | --- | --- |
| 1 | N | 13 | N | Y | N | Irregular and focally atypical keratinocytic proliferation occurring in association with ulcer with fibrosing granulation tissue. | N |
| 2 | N | 11 | N | N | N | - | N |
| 3 | N | 14 | N | N | N | - | N |
| 4 | N | 7 | N |  | N | - | Y |
| 5 | Y | 17 | N | Y | N | Necrotizing neutrophilic inflammation and abscess. Pseudoepitheliomatous hyperplasia and other reactive changes present in acral skin. | N |
| 6 | N | 16 | N | N | N | - | N |
| 7 | N | 9 | N | N | N | - | Y |
| 8 | N | 7 | N | Y | N | Fibrinous exudate, acute ulceration, and granulation tissue with focal foreign body giant cell reaction and reactive endothelial atypia | Y |
| 9 | Y | 16 | N | Y | Y | Ulcerated skin with intense intradermal neutrophilic inflammation, abscess, and necrosis. | N |
| 10 | N | 14 | N | Y | Y | Mild acanthosis and dermal edema with mixed infiltrate of neutrophils, eosinophils, lymphocytes, histiocytes, and plasma cells. | N |
| 11 | N | 16 | N | N | N | - | N |
| 12 | N | 18 | Y | Y | Y | Suppurative inflammation with surrounding lymphoplasmacytic infiltrate. | N |
| 13 | N | 10 | N | Y | N | Dense neutrophilic infiltrate in the deep dermis and superficial subcutis with focal necrosis and macrophages and few multinucleated giant cells. | N |
| N14 | N | 15 | N | Y | N | Ulceration with underlying fibrosis, granulation tissue and vasculitis changes. No classic histologic evidence of pyoderma gangrenosum is seen. | N |
| 15 | N | 16 | N | Y | N | Ulceration, exuberant suppurative inflammation within the dermis and subcutaneous tissue. | N |
| 16 | N | 14 | N | Y | N | Chronic calciphylaxis occurring in a background of venous insufficiency with supervening metastatic calcification of the subcutis and significant plasmacytosis. | N |
| 17 | N | 11 | N | Y | Y | Ulcer, suppurative and granulomatous dermal/subcutaneous inflammation and fat necrosis | Y |
| 18 | N | 15 | N | N | N | - | N |
| 19 | N | 18 | N | Y | Y | Ulceration, extensive neutrophilic inflammation and necrosis. | N |
| 20 | Y | 18 | Y | Y | Y | Reactive epidermal changes, edema, hemorrhage, mixed inflammation and scar. Abundant neutrophils noted. | N |
| 21 | Y | 19 | N | Y | Y | Mixed neutrophilic inflammation with granulomas and scar. | N |
| 22 | Y | 19 | Y | Y | Y | Hemorrhage and neutrophilic infiltration of superficial/deep reticular dermis and subcutis. | N |
| 23 | N | 16 | N | N | N | - | N |
| 24 | N | 18 | Y | Y | Y | Ulcer with associated granulation changes. Negative for malignancy. | N |
| 25 | N | 18 | N | N | N | - | N |
| 26 | N | 11 | N | N | N | - | N |
| 27 | N | 15 | N | N | N | - | N |
| 28 | N | 11 | N | N | N | - | N |
| 29 | N | 14 | N | Y | N | Ulcer with necrotizing granulomatous dermal and subcutaneous inflammation, weak perivascular C3, and fibrinogen staining. | N |
| 30 | N | 15 | N | N | N | - | N |
| 31 | N | 19 | Y | N | N | - |  |
| 32 | N | 8 | N | N | N | - | Y |
| 33 | N | 17 | N | N | N | - | N |
| 34 | N | 0 | N | N | N | - | Y |
| 35 | N | 19 | Y | N | N | - |  |
| 36 | N | 17 | Y | Y | N | Fibroadipose tissue with fat necrosis, suppurative granulomatous and lymphocytic inflammation. | N |
| 37 | N | 16 | N | Y | N | No neutrophilic infiltration or ulceration suggestive of chronic venous stasis. | N |
| 38 | N | 17 | Y | Y | N | Epidermal acanthosis, dermal edema, perivascular lymphocytic and plasma cell inflammation, focal fat necrosis. | N |
| 39 | N | 16 | N | N | N | - | N |
| 40 | N | 18 | N | N | N | - | N |
| 41 | N | 15 | N | Y | Y | Acanthosis with overlying hyperkeratosis, parakeratosis, dermal edema and slight perivascular mixed inflammation. | N |
| 42 | N | 19 | Y | N | N | - | N |
| 43 | N | 10 | N | N | N | - | N |
| 44 | N | 19 | Y | Y | N | Non-specific chronic active ulcer fragment. | N |
| 45 | N | 13 | N | N | N | - | N |
| 46 | N | 10 | N | Y | N | Granulomatous and neutrophilic septal and focally lobular panniculitis. | N |
| 47 | N | 14 | N | Y | N | Dermal septal fibrosis with increased small vessels in the subcutis possibly exhibiting thickened intima. | N |
| 48 | N | 11 | N | Y | N | Sparse neutrophils, mixed with lymphocytes and histiocytes. | N |
| 49 | Y | 18 | Y | Y | Y | Nodular infiltrate of neutrophils, mixed with lymphocytes and histiocytes. | N |
| 50 | N | 18 | N | N | N | - | N |
| 51 | N | 17 | Y | Y | N | Ulcer with dermal granulation tissue. | N |
| 52 | N | 15 | N | N | N | - | N |
| 53 | N | 16 | N | N | N | - | N |
| 54 | N | 16 | N | Y | N | Extensive superficial and deep dermal necrosis with edema, basophilic necrotic changes, fat necrosis, with mixed infiltrate of neutrophils, lymphocytes, histiocytes and scattered eosinophils. | N |
| 55 | N | 11 | N | Y | N | BCC | Y |
| 56 | N | 16 | N | N | N | - | N |
| 57 | N | 16 | N | N | N | - | N |
| 58 | N | 16 | Y | N | N | - | N |

*Note:Histological findings extracted from pathology report narrative. Negative results for PAS, fungal, and other special stains were not included in the report.*
